# Imaging of ROR1 expression in tumors using radiolabeled affibody molecules

**DOI:** 10.1007/s00259-026-08106-8

**Published:** 2026-08-01

**Authors:** Cornelia Westerberg, Tianqi Xu, Maria Marth, Moeen Ud-Din, Russol Abushaia, Tilde Hagelin, Stefan Ståhl, Anna Orlova, Vladimir Tolmachev, John Löfblom, Anzhelika Vorobyeva

**Affiliations:** 1 Department of Protein Science, School of Engineering Sciences in Chemistry, Biotechnology and Health, KTH Royal Institute of Technology, 100 44 Stockholm, Sweden; 2 Department of Immunology, Genetics and Pathology, Uppsala University, 751 85 Uppsala, Sweden; 3 Department of Medicinal Chemistry, Uppsala University, 751 23 Uppsala, Sweden

**Keywords:** ROR1, Radionuclide imaging, Affibody, MDA-MB-468

## Abstract

**Purpose:**

Receptor tyrosine kinase-like orphan receptor 1 (ROR1) is an attractive molecular target for anti-tumor therapies undergoing late-stage clinical development. Quantitative imaging of ROR1 expression could enable identification of patients likely to respond to such treatments. This study aimed to assess the feasibility of specific *in vivo* imaging of ROR1 using radiolabeled affibody molecules.

**Methods:**

The affibody molecule ZROR1:A10, with high affinity for human (4 nM) and murine (2 nM) ROR1, was labeled with indium-111 using a DOTA chelator. *In vitro* binding specificity, affinity, and cellular processing were evaluated using a panel of ROR1-expressing cell lines. The affibody [^111^In]In-DOTA-Zcov19s, which does not bind ROR1, served as a nonspecific control. The biodistribution of [^111^In]In-DOTA-ZROR1:A10 and [^111^In]In-DOTA-Zcov19s was measured in immunodeficient mice bearing ROR1-positive tumors, with ROR1-negative Ramos xenografts used as an additional specificity control.

**Results:**

[^111^In]In-DOTA-ZROR1:A10 bound specifically to ROR1-expressing cells, with significantly higher uptake than [^111^In]In-DOTA-Zcov19s. The apparent equilibrium dissociation constant for ROR1 binding *in vitro* was 1–4 nM. Tumor uptake of [^111^In]In-DOTA-ZROR1:A10 in MDA-MB-468 xenografts was saturable and significantly (p < 0.05) higher than in Ramos tumors, as well as higher than uptake of the control affibody. At 4 h post‑injection of 1 µg (50 µg/kg), tumor‑to‑blood, tumor‑to‑bone, and tumor‑to‑muscle ratios were 14 ± 6, 19 ± 6, and 11 ± 1, respectively. Micro-SPECT/CT imaging clearly visualized ROR1‑positive tumors and discriminated them from ROR1‑negative xenografts.

**Conclusion:**

The preclinical evaluation shows that visualization of ROR1-expressing tumors using a radiolabeled affibody molecule is feasible.

**Supplementary Information:**

The online version contains supplementary material available at https://doi.org/10.1007/s00259-026-08106-8.

## Introduction

Receptor tyrosine kinase-like orphan receptor 1 (ROR1) is a type I transmembrane receptor belonging to the ROR family, which also includes ROR2. The structure of the ROR1 oncofetal protein includes an extracellular immunoglobulin-like domain, a Frizzled-like cysteine‑rich domain, a Kringle domain, and an intracellular region with a tyrosine kinase domain, serine/threonine-rich domains, and a proline‑rich domain. These features enable ROR1 to function in multiple signaling pathways that regulate cellular behavior [1].

In mammals, ROR1 acts primarily as a receptor or co‑receptor for non‑canonical Wnt5a-mediated signaling, contributing to the regulation of cell polarity, migration, proliferation, and differentiation. These processes are essential during early embryogenesis, organogenesis, and tissue morphogenesis. ROR1 is highly expressed in developing tissues, but its expression is restricted mainly to specific stem or progenitor cell populations in most adult tissues [2]. ROR1 expression might be induced in adult tissues in response to injury to support repair [3]. More recent study demonstrated cell-surface expression of ROR1 expression in selected adult human tissues, including parathyroid glands, pancreatic islets, and regions of the upper gastrointestinal tract [4].

Aberrant ROR1 re‑expression is observed in hematologic [5, 6], breast [7], ovarian [8], and pancreatic malignancies [9]. ROR1 enhances tumor progression by inhibiting apoptosis, promoting epithelial-mesenchymal transition, potentiating EGFR signaling, and supporting cancer stem‑cell survival, migration, invasion, and chemoresistance. The restricted expression in normal tissues, combined with robust pathological upregulation, makes ROR1 an attractive therapeutic target [1, 10, 11].

Multiple ROR1-targeting drugs, including antibody‑drug conjugates (ADCs), monoclonal antibodies, bispecific T‑cell engagers, and CAR‑T cells, are currently under clinical evaluation [12]. Zilovertamab vedotin, an ADC carrying MMAE directed against ROR1, demonstrated an impressive response rate in patients with mantle cell and diffuse large B-cell lymphomas already in a Phase I clinical study [13]. However, the objective response rate to zilovertamab vedotin in solid tumors was disappointingly low (1 of 17) [14]. Notably, the trial did not select participants based on ROR1 expression, and ROR1 expression was observed in only 3 of 17 participants. The investigators concluded that prospective selection of participants or tumor subtypes based on ROR1 expression could result in higher antitumor activity [14]. Stratification of patients according to ROR1 expression level is essential, as there is substantial interpatient heterogeneity of expression in solid tumors [4, 15]. Moreover, hypoxia may induce a decrease in ROR1 expression in, for example, melanoma [16]. The expression level of ROR1 may change in response to therapy. For example, expression may be increased after treatment of breast cancer with the antibody–drug conjugate T-DM1 [17] or neoadjuvant chemotherapy of ovarian cancer [18]. Thus, the success of further development and application of ROR1-targeting therapeutics may depend on successful *in vivo* measurement and real-time monitoring of its expression in tumors.

Radionuclide imaging of prostate-specific membrane antigen (PSMA) and somatostatin receptor 2 (SSTR2) expression levels was decisive in enabling successful precision radionuclide therapy in prostate and neuroendocrine tumors [19]. This development triggered substantial interest in radionuclide imaging-guided targeted therapy for other malignancies. The approach was subsequently expanded to visualization of therapeutic target expression using non-radiolabeled antibodies [20]. Several imaging probes based on radiolabeled monoclonal antibodies [21] or de novo selected ROR1-binding peptides [22, 23] have been preclinically evaluated as potential radiotracers for *in vivo* visualization of ROR1.

Affibody molecules are compact (6–7 kDa) affinity proteins engineered based on a three helical bundle scaffold [24]. The use of combinatorial libraries enables the selection of affibody molecules that bind with high affinity to cell-surface proteins associated with malignant transformation [25–27]. Coupling affibody molecules with gamma- or positron-emitting radionuclides provides targeting probes for *in vivo* molecular imaging. Several preclinical investigations have demonstrated that radiolabeled affibody molecules allow sensitive tumor visualization within 2–3 h after injection, compared with radiolabeled antibodies, which require imaging 2–3 days after injection [28]. Clinical evaluations of an affibody molecule targeting human epidermal growth factor receptor 2 (HER2) have shown that injections are safe and well tolerated, without inducing the formation of neutralizing antibodies in patients [29]. Data from early-stage clinical trials confirmed the clinical utility of affibody-based imaging. For example, quantitative PET imaging of HER2 expression in breast cancer using [^68^ Ga]Ga-labeled affibody molecule ABY-025 enabled prediction of early metabolic response to HER2-targeted therapy with anti-HER2 monoclonal antibody trastuzumab [30].

Given the favorable pharmacokinetics and clinical performance of radiolabeled affibody molecules, this class of targeting probes represents a promising approach for non‑invasive, quantitative imaging of therapeutically relevant tumor targets. In this study, we therefore aimed to evaluate the feasibility of ROR1 imaging using a radiolabeled affibody molecule. To this end, a high-affinity ROR1-binding affibody was selected, site-specifically radiolabeled, and evaluated preclinically as a candidate imaging probe.

## Materials and methods

A detailed description of the Materials and Methods is provided in the Supplementary Information, including affibody design, production and characterization, radiolabeling procedures, in vitro assays, and animal studies.

## Results

### Selection and identification of ROR1-binding affibody molecules

**Fig. 1:**
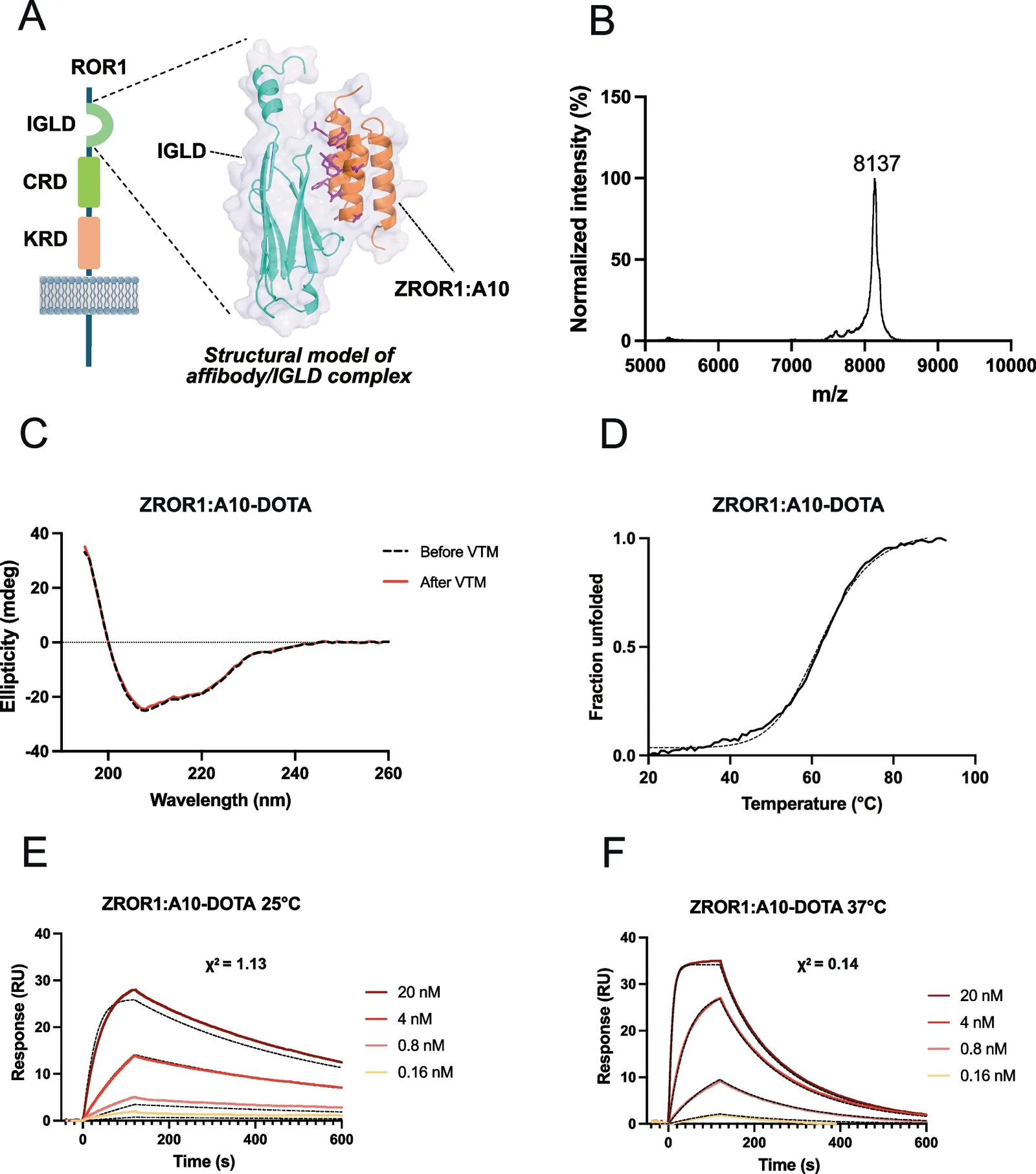
**A** Schematic representation of the ROR1 domain architecture and structural model of affibody binding. ROR1 is shown as a membrane-anchored receptor comprising an extracellular immunoglobulin-like domain (IGLD), cysteine-rich domain (CRD), and kringle domain (KRD). The structural model illustrates the interaction between the ROR1 IGLD and the affibody ZROR1:A10, predicted using AlphaFold3. The affibody binds to a surface-exposed epitope on the IGLD, as indicated by the mapped interface residues. **B** Mass spectra from MALDI-TOF analysis of candidate ZROR1:A10 **C** CD spectra of the conjugated candidate ZROR1:A10-DOTA before and after heat denaturation. **D** Heat denaturation of ZROR1:A10-DOTA with the fitted curve (dashed) used to calculate the T_m_. **E** Kinetic evaluation of ZROR1:A10-DOTA interacting with recombinant ROR1 immobilized on the SPR chip surface, performed at 25 °C. Colored curves represent experimental data and dotted lines represent the global fit using a 1:1 Langmuir binding model (χ^2^ = 1.13). **F** Kinetic evaluation of ZROR1:A10-DOTA interacting with recombinant ROR1 immobilized on the SPR chip surface, performed at 37 °C. Colored curves represent experimental data and dotted lines represent the global fit using a 1:1 Langmuir binding model (χ^2^ = 0.14)

Affibody molecules binding human ROR1 were selected from an *Escherichia coli* surface display affibody library comprising approximately 10^11^ variants [26, 27]. Details of the selection strategy and protein engineering process are beyond the scope of the present study and will be reported separately. Briefly, selections were performed against recombinant biotinylated human ROR1 ectodomain, as described previously [25–27]. Binding of soluble candidates to recombinant ROR1 was evaluated by surface plasmon resonance (Supplementary Fig. 1) and based on its binding profile, including cross-reactivity to mouse ROR1 (Supplementary Table 1), clones A10 and A50 (hereafter denoted ZROR1:A10 and ZROR1:A50) were selected for an initial cell-binding screen using the ROR1-expressing cell lines MDA-MB-468, HT29, and SKOV-3. Although the differences in affibody binding between the cell lines were modest, the observed binding shifts were consistent with the relative cell surface ROR1 staining obtained using a ROR1-specific monoclonal antibody (Supplementary Fig. 2). ZROR1:A50 displayed a tendency to aggregate during production and handling, and ZROR1:A10 was therefore selected for further development as a molecular imaging probe targeting ROR1. Its amino acid sequence is shown in Supplementary Fig. 3. Structural models of the interaction between ZROR1:A10 and the extracellular region of human ROR1 (UniProt: Q01973) were generated using AlphaFold3 (Fig. 1A, Supplementary Fig. 4). The models predicted binding of ZROR1:A10 to the Ig-like domain (IGLD), the most distal domain of ROR1, with high interface confidence (ipTM ≈ 0.9). No interactions were predicted with other extracellular domains (Supplementary Table 2).

### Production and characterization of DOTA-conjugated affibody molecules

ZROR1:A10, and a negative control affibody with non-related specificity (Zcov19S) were subcloned into a (HE)_3_-Z-cys construct. The hexa-histidine-glutamine tag in the N terminus has previously been shown to reduce hepatic uptake of affibodies [31], and the cysteine in the C terminus was included for site-specific conjugation of DOTA chelator, using maleimide chemistry. Both ZROR1:A10 and the negative control affibody were produced, followed by DOTA conjugation and purification by HPLC (Supplementary Fig. 5). Molecular masses for non-conjugated and conjugated proteins were determined by mass spectrometry and were in agreement with the theoretical values (Fig. 1B, Supplementary Fig. 6). Variable‑temperature measurements using circular dichroism spectroscopy showed that DOTA-conjugated ZROR1:A10 had an alpha helical secondary structure and a melting temperature of 62 °C and refolded well after thermal denaturation (Fig. 1C, D). Surface plasmon resonance analysis demonstrated that DOTA-conjugated ZROR1:A10 retained high affinity for ROR1, with an association rate constant (kₐ) of 2.0 ± 0.3 × 10^6^ M⁻^1^ s⁻^1^ and a dissociation rate constant (k_d_) of 1.6 ± 0.3 × 10⁻^4^ s⁻^1^, corresponding to an equilibrium dissociation constant (K_D_) of 0.8 ± 0.2 nM at 25 °C (Fig. 1E, F), while Zcov19-DOTA did not show binding to ROR1 (Supplementary Fig. 7).

### Radiolabeling and *in vitro* stability

**Fig. 2:**
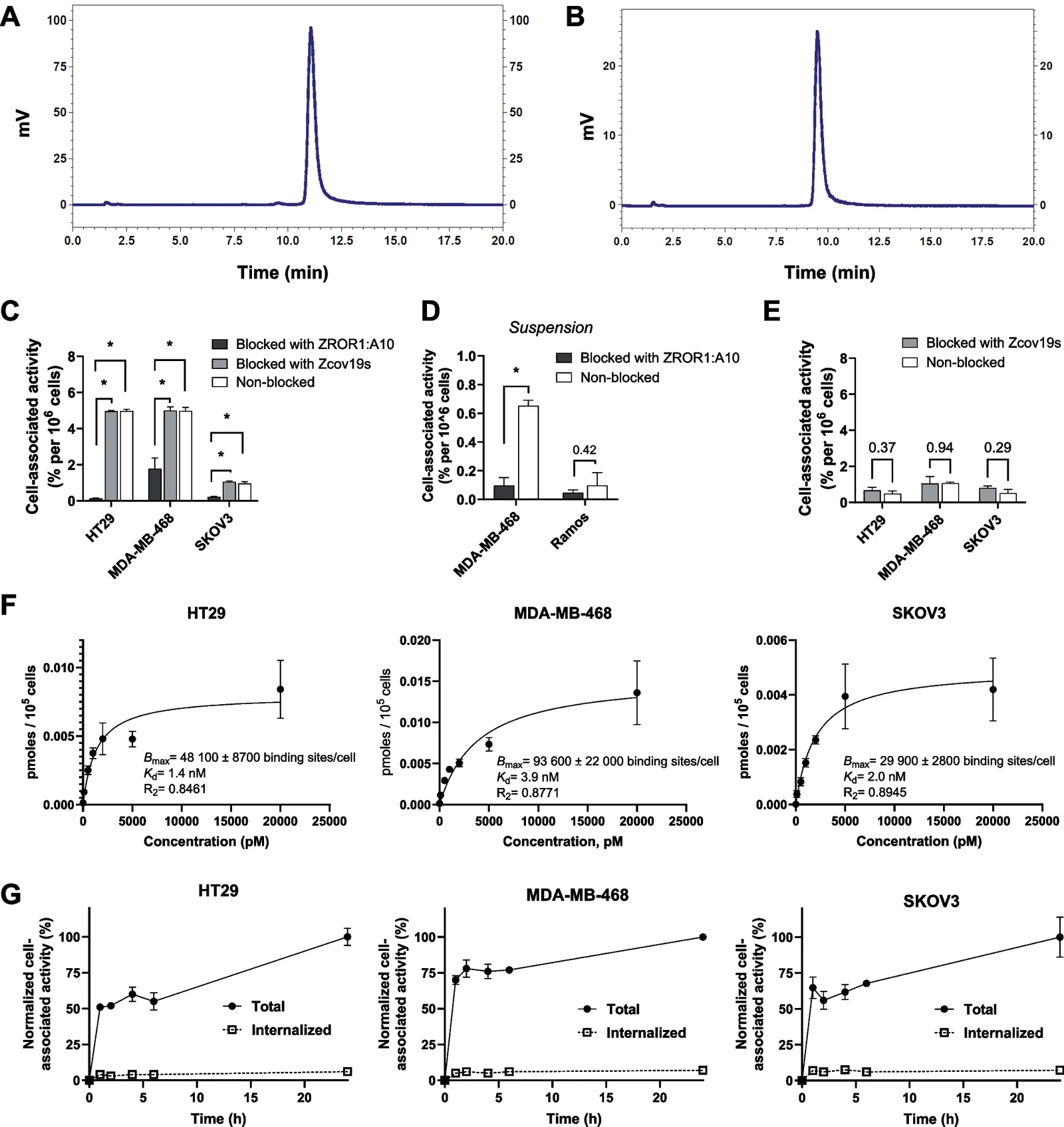
*In vitro* characterization of the radiolabeled ROR1‑binding affibody [^111^In]In‑ZROR1:A10. Radio‑HPLC analysis of [^111^In]In‑ZROR1:A10 (**A**) and the nonspecific control affibody [^111^In]In‑Zcov19s (**B**). *In vitro* binding specificity of [^111^In]In‑ZROR1:A10 (**C**) and [^111^In]In‑Zcov19s (**E**) in ROR1‑expressing cancer cell lines following incubation with 1 nM radiolabeled affibody. Binding specificity was assessed by receptor blocking using a 1000‑fold molar excess of the corresponding non‑labeled affibody. Validation of ROR1-specific binding using ROR1-negative Ramos cells as a negative control (**D**). Quantification of ZROR1:A10-accessible ROR1 binding sites on MDA‑MB‑468, HT‑29, and SKOV3 cells determined by saturation binding assay (**F**). Cellular processing and internalization of [^111^In]In‑DOTA‑ZROR1:A10 in HT29, MDA‑MB‑468, and SKOV3 cells (**G**). Data are presented as mean ± standard deviation (*n* = 3)

Radiolabeling of DOTA-conjugated ZROR1:A10 and Zcov19s with indium-111 resulted in good radiochemical yields. After size-exclusion purification, high radiochemical purity was obtained. Radio-HPLC analysis confirmed high purity (> 99%) for both radiolabeled affibody molecules (Fig. 2A, B). The radiolabel was stable, withstanding challenge by a large excess of EDTA for up to 24 h (Table 1).

**Table 1:** Radiolabeling and *in vitro* stability of ^111^In-labeled DOTA-ZROR1:A10 and DOTA-Zcov19s affibody molecules

	RCY	RCP	Stability protein-associated activity (%)					
			1 h		4 h		24 h	
			× 1000 EDTA	PBS	× 1000 EDTA	PBS	× 1000 EDTA	PBS
[^111^In]In-DOTA-ZROR1:A10	85 ± 5 (*n* = 3)	99 ± 1 (*n* = 3)	99 ± 0 (*n* = 2)	99 ± 0 (*n* = 2)	99 ± 0 (*n* = 2)	99 ± 0 (*n* = 2)	98 ± 0 (*n* = 2)	98 ± 0 (*n* = 2)
[^111^In]In-DOTA-Zcov19s	42 (*n* = 1)	100 (*n* = 1)	99 ± 0 (*n* = 2)	100 ± 0 (*n* = 2)	98 ± 1 (*n* = 2)	100 ± 0 (*n* = 2)	97 ± 0 (*n* = 2)	99 ± 0 (*n* = 2)

### *In vitro* evaluation of [^111^In]In-ZROR1:A10

The specificity of [^111^In]In-ZROR1:A10 binding to ROR1-expressing human cancer cell lines was tested by receptor pre-blocking with a 1000-fold molar excess of either unlabeled ZROR1:A10 or the nonspecific control Zcov19s. Binding of [^111^In]In-ZROR1:A10 was significantly (*p* < 0.05) reduced in the presence of excess non-labeled ZROR1:A10, whereas pre-incubation with Zcov19s had no effect (Fig. 2C). To further confirm the specificity of [^111^In]In-ZROR1:A10 binding, ROR1-negative Ramos cells were evaluated as a negative control. No significant difference was observed between blocked and non-blocked conditions in Ramos cells (*p* = 0.4198), and the residual cell-associated activity was comparable to that observed in blocked MDA-MB-468 samples, indicating nonspecific background binding (Fig. 2D).

Binding of the control affibody molecule [^111^In]In-Zcov19s was significantly (*p* < 0.05) lower than that of the ROR1-binding [^111^In]In-ZROR1:A10, and pre-saturation with unlabeled Zcov19s did not affect cell-bound activity (Fig. 2E).

Results of the saturation binding assay are presented in Fig. 2F. The B_max_ values obtained for [^111^In]In-ZROR1:A10 binding corresponded to 93600 ± 22000, 48100 ± 8700, and 29900 ± 2800 ZROR1:A10-accessible binding sites per cell for MDA-MB-468, HT-29, and SKOV3 cells, respectively.

Cellular processing and internalization of [^111^In]In-DOTA-ZROR1:A10 were evaluated in MDA-MB-468, HT29, and SKOV3 cells over 24 h. A similar binding pattern was observed for all cell lines (Fig. 2G) with rapid binding during the first hour followed by a slower increase up to 24 h. Internalization occurred slowly in all cell lines. At 24 h, the internalized fraction was 6.6 ± 0.2% in MDA-MB-468 cells, 6.1 ± 0.1% in HT29 cells, and 7 ± 1% in SKOV3 cells.

### *In vivo* evaluation of [^111^In]In-ZROR1:A10

**Fig. 3:**
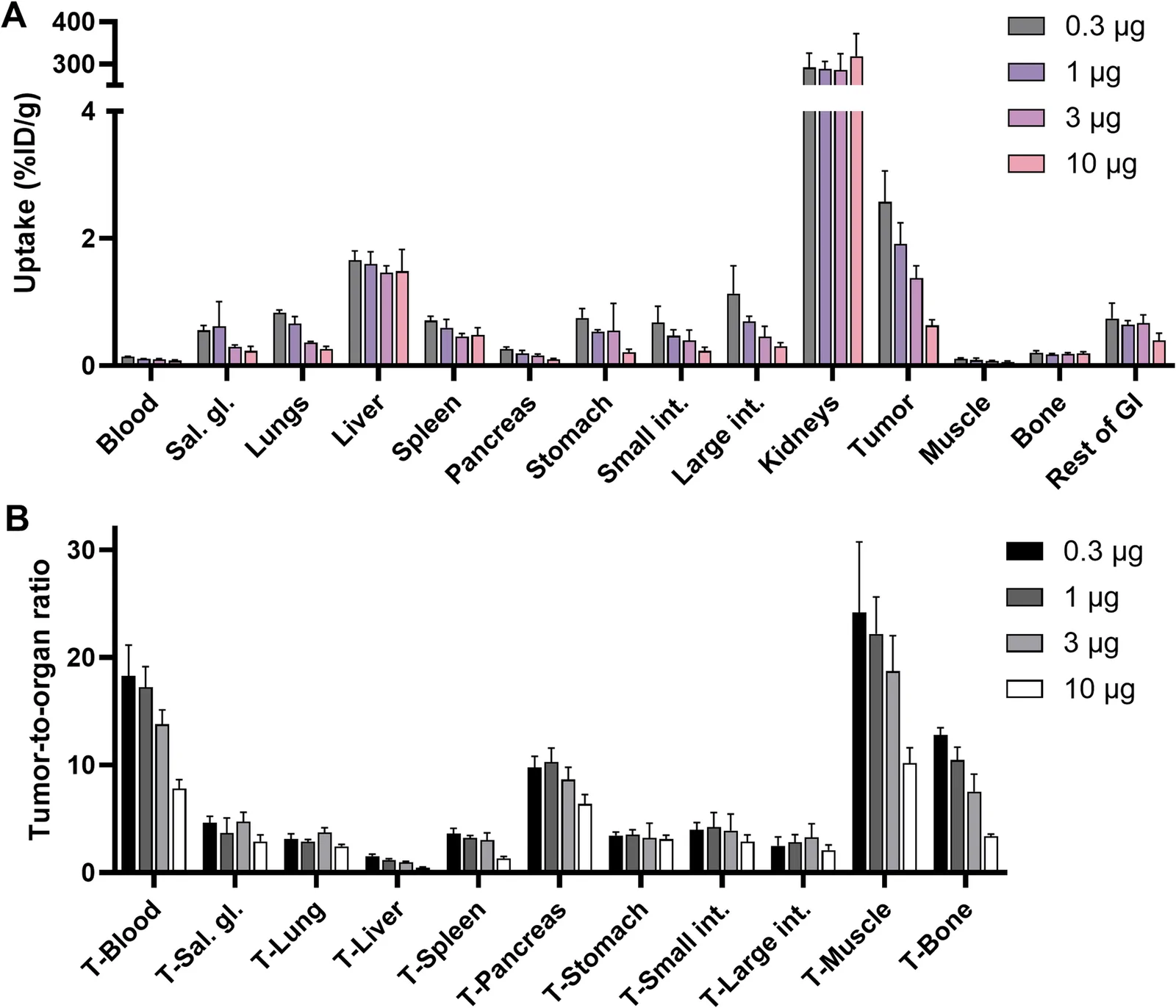
Dose-dependent biodistribution and tumor-to-organ ratios of [^111^In]In-ZROR1:A10 in Balb/c nu/nu mice bearing MDA-MB-468 xenografts. **A** Biodistribution at 4 h p.i. (0.3–10 μg) (*n* = 3–4, mean ± SD); **B** Tumor-to-organ ratios at 4 h p.i

The biodistribution of [^111^In]In-DOTA-ZROR1:A10 in mice bearing MDA-MB-468 xenografts (Fig. 3) exhibited a pattern characteristic for affibody molecules. Blood activity concentrations were low, ranging from 0.08 to 0.14%ID/g. The highest total organ uptake was observed in the kidneys, where approximately 80% of the injected activity (%ID) accumulated. Hepatic uptake was substantially lower, less than 2% of the injected activity (%ID) and did not depend on the injected protein mass, as no significant differences were observed between groups (*p* > 0.05, one-way ANOVA test with Bonferroni correction). When normalized to tissue weight, these values corresponded to approximately 300%ID/g and 2%ID/g for kidneys and liver, respectively. No impact of injected mass was observed on uptake in the stomach, muscles, or bone. In contrast, uptake in the salivary glands, lungs, spleen, pancreas, and large intestine decreased with increasing injected mass of DOTA-ZROR1:A10, consistent with dose-dependent saturation of accessible binding sites (self-blocking effect). Statistically significant differences were observed between the 0.3 µg and 10 µg injected protein groups for blood, lungs, spleen, pancreas, small intestine, large intestine, tumor, and muscle uptake (Fig. 3A).

A clear trend toward reduced tumor uptake was observed with increasing injected mass of [^111^In]In-DOTA-ZROR1:A10. While there was no significant difference in tumor uptake between injections of 0.3 and 1 µg, further dose escalation led to a significant decrease in tumor accumulation. Consistently, tumor‑to‑organ ratios did not differ significantly between the 0.3 and 1 µg groups, whereas injection of higher protein masses resulted in significantly reduced ratios (Fig. 3B). This decrease was most pronounced for tumor‑to‑blood, tumor‑to‑muscle, and tumor‑to‑bone ratios.

**Fig. 4:**
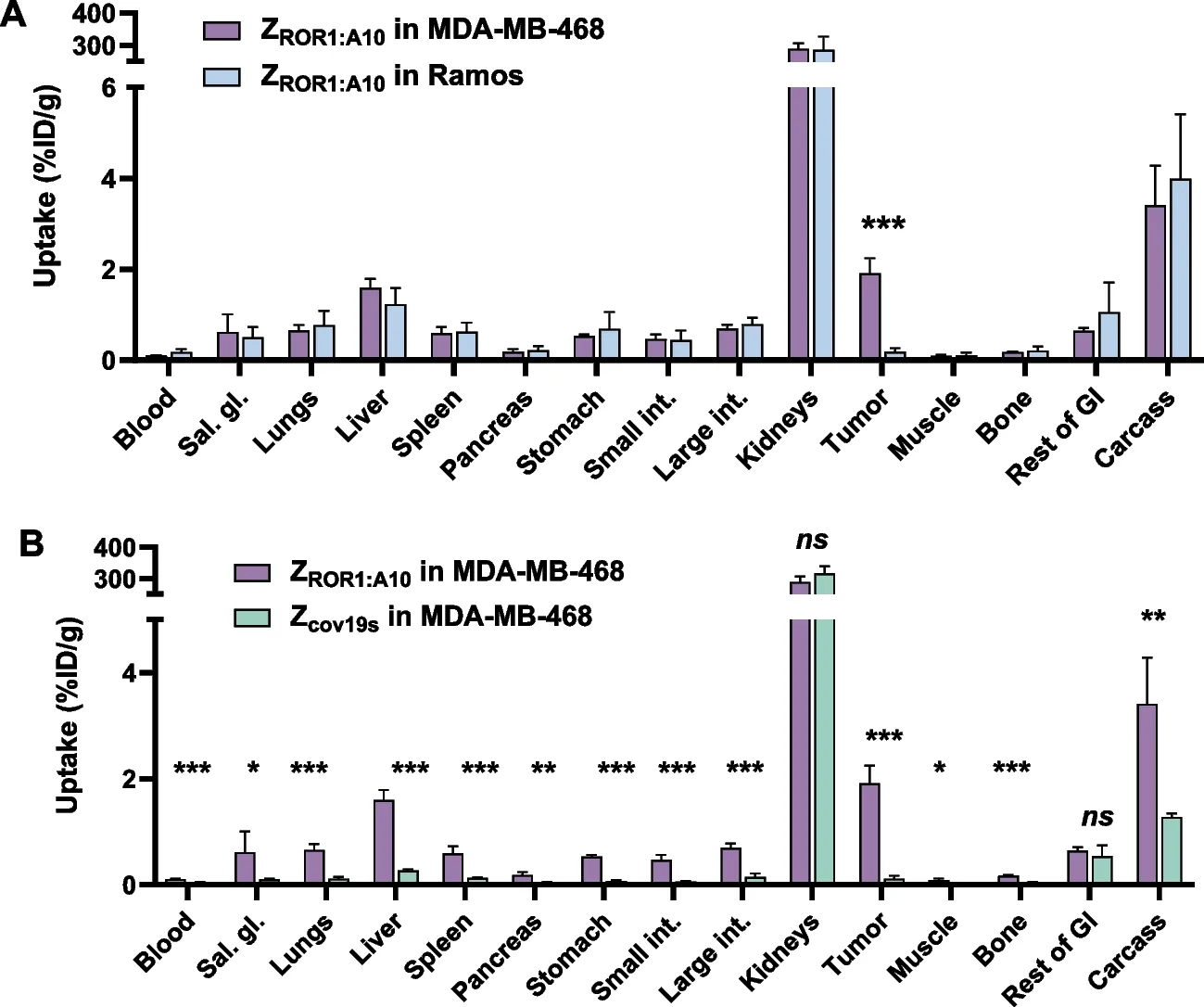
*In vivo* targeting specificity of [^111^In]In-ZROR1:A10 (1 μg) in Balb/c nu/nu mice bearing ROR1-expressing MDA-MD-468 xenografts 4 h p.i. (*n* = 4, mean ± SD). A. Ramos xenografts were used as a ROR1-negative control. B. Comparison with [^111^In]In-Zcov19s

To assess ROR1‑mediated uptake, biodistributions of [^111^In]In‑DOTA‑ZROR1:A10 in mice bearing ROR1‑negative Ramos xenografts and of the nonspecific control [^111^In]In‑Zcov19s in mice bearing MDA‑MB‑468 xenografts were evaluated (Fig. 4). Tumor uptake of [^111^In]In‑ZROR1:A10 in Ramos xenografts was ten‑fold lower than in MDA‑MB‑468 tumors (*p* < 0.0005), while uptake in normal tissues was comparable. Marked differences were observed between the biodistributions of ^[111^In]In‑ZROR1:A10 and [^111^In]In‑Zcov19s. The kidney was the only organ showing similar uptake of both tracers, whereas uptake of [^111^In]In‑Zcov19s in all other organs was significantly lower (typically *p* < 0.005). Uptake of [^111^In]In‑Zcov19s in ROR1‑positive MDA‑MB‑468 tumors was 15‑fold lower than that of [^111^In]In‑ZROR1:A10.

**Fig. 5:**
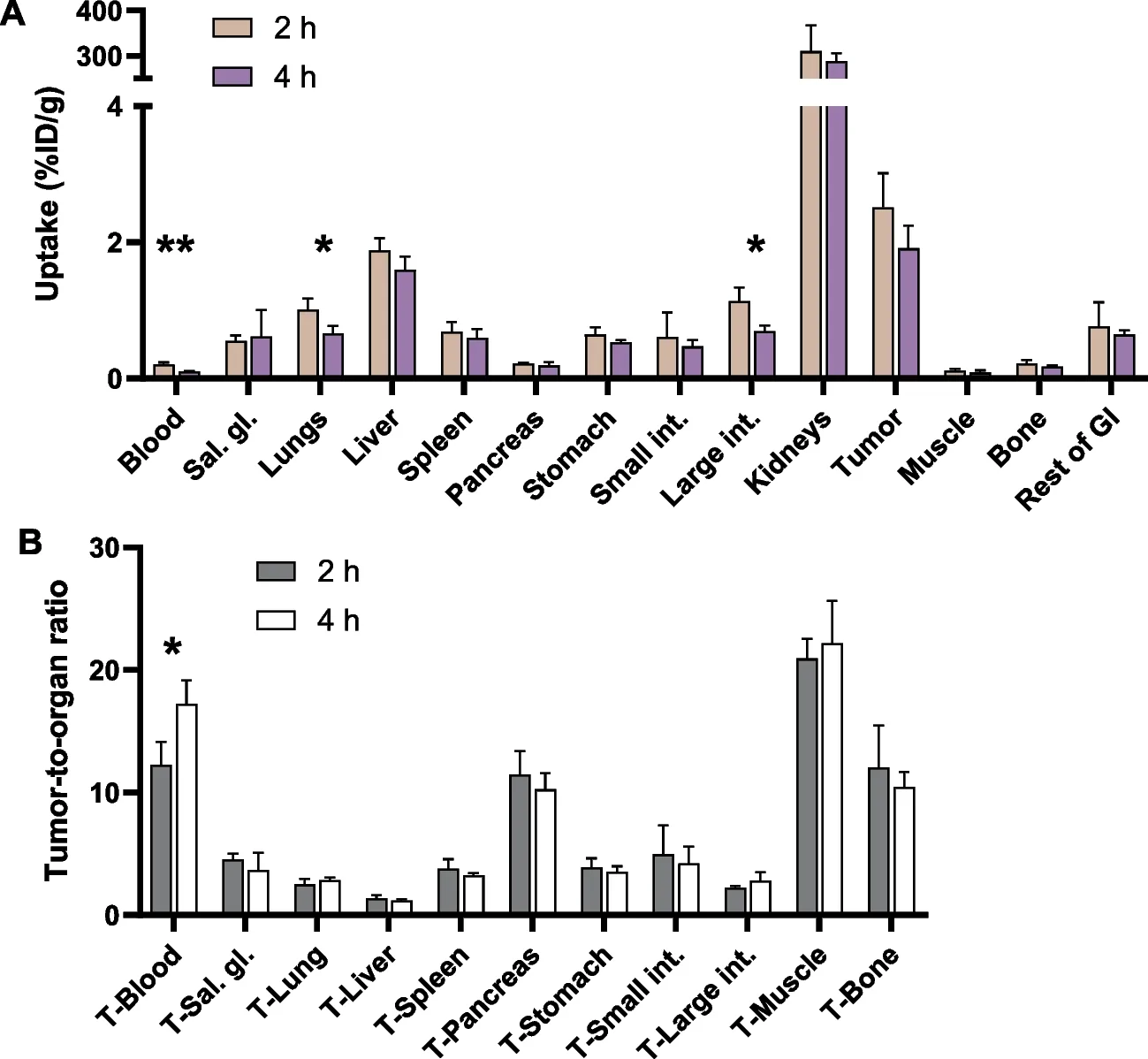
Comparison of biodistribution (**A**) and tumor-to-organ ratios (**B**) at 2 and 4 h after injection of [^111^In]In-ZROR1:A10 (1 μg) in mice bearing ROR1-expressing MDA-MD-468 xenografts. Data are presented as mean ± SD (*n* = 4)

To evaluate time‑dependent tracer clearance, biodistribution of [^111^In]In-ZROR1:A10 in mice bearing MDA-MB-468 xenografts was additionally measured at 2 h post-injection (Fig. 5). Compared with 2 h, uptake at 4 h was significantly (*p* < 0.05) reduced only in blood, lungs, and the large intestine wall. Although tumor uptake showed a trend toward lower values at 4 h post-injection, this difference did not reach statistical significance in the present study. Tumor‑to‑blood ratios increased significantly with time (*p* < 0.05), while other tumor‑to‑organ ratios did not change significantly.

**Fig. 6:**
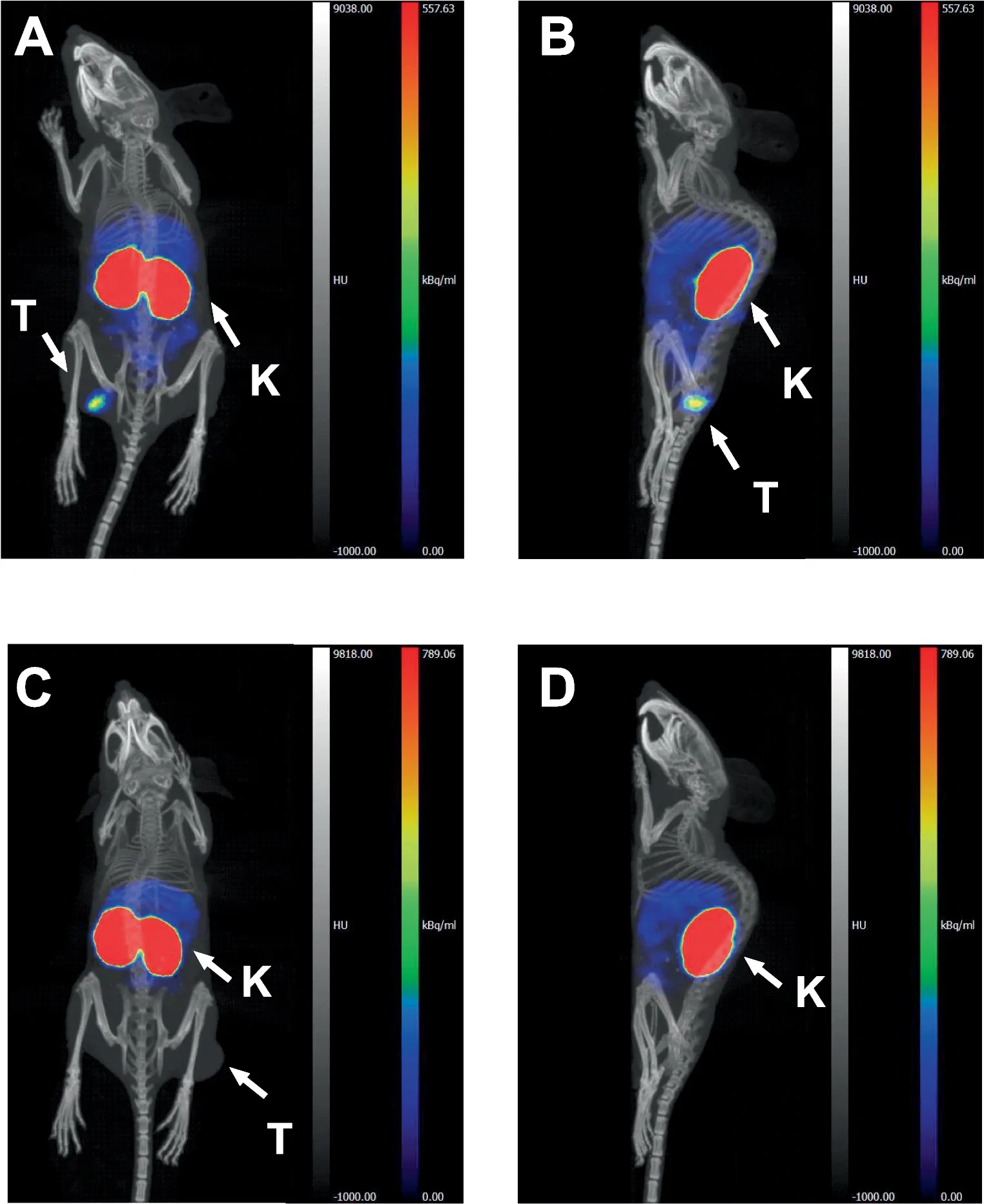
Coronal (**A**, **C**) and sagittal (**B**, **D**) fused SPECT/CT maximum-intensity projections of mice bearing ROR1-positive MDA-MB-468 (**A**, **B**) and ROR1-negative Ramos (**C**, **D**) human xenografts. Arrows indicate tumors (T) and kidneys (K)

NanoSPECT/CT imaging (Fig. 6) confirmed the *ex vivo* biodistribution data. MDA-MB-468 xenograft was clearly visualized, with the kidneys being the only organs showing higher uptake. Uptake in ROR1-negative Ramos tumor was not visible using the same scale setting.

## Discussion

Targeted therapies can reduce the toxicity of cancer treatment by selectively directing therapeutic agents to malignant tissue. However, for such therapies to be effective, sufficient target expression is needed and patients should be selected accordingly to avoid overtreatment. Biopsy-based diagnostic methods are commonly used for this purpose, but may have limited reliability due to heterogeneity of target expression, potentially resulting in failure to detect target-positive metastases or misclassification of target-negative metastases. Radionuclide-based visualization of target expression offers a non-invasive approach to overcome this limitation [32, 33]. Current strategies for the development of companion imaging diagnostics include radiolabeled variants of therapeutic antibodies (immunoPET), radiolabeled natural receptor ligands, and radiolabeled engineered scaffold proteins. ImmunoPET using large intact therapeutic antibodies usually requires several days to achieve sufficient accumulation in tumors and clearance from non-specific compartments for high-contrast imaging [28]. Radiolabeled analogues of short natural peptide ligands, such as somatostatin, bombesin, neurotensin, or substance P, can overcome this limitation and allow for imaging at substantially earlier time points [34]. However, the natural ligand of ROR1, Wnt5a [35], is a relatively large (45 kDa [36]), and structurally complex protein, making it less suitable as an imaging probe due to slow extravasation and localization in tumors. In addition, Wnt proteins contain multiple essential disulfide bonds [37], limiting the options for site-specific labeling, and use of reducing conditions. Another strategy for tracer development is based on engineered non-immunoglobulin scaffold proteins, such as affibody molecules, which combine high-affinity binding with a small molecular size [38]. The successful application of the affibody scaffold for the development of HER2-specific imaging probes has provided a strong rationale for the exploration of affibody‑based imaging agents also for other targets and disease areas.

Here, the DOTA-conjugated binder (HE)₃-ZROR1:A10-DOTA demonstrated a high affinity for human ROR1 (K_D_ = 0.8 nM) (Fig. 1), which should be sufficient for radionuclide imaging purposes [39]. This value indicated a somewhat higher apparent affinity than that observed during the affinity screening of the unconjugated affibody used for lead selection. In addition to differences in protein format and DOTA conjugation, the observed difference in K_D_ values could be attributed to factors such as production batch variability in both the candidate and the recombinant ROR1 protein, as well as differences in sensor chip immobilization and experimental conditions. The observed difference is therefore likely to reflect normal experimental variation rather than an affinity improvement resulting from DOTA conjugation.

Importantly, ZROR1:A10 is cross-reactive for murine ROR1, making the mouse model relevant not only for preclinical evaluation of tumor-targeting specificity but also for assessment of potential interactions with physiological ROR1 expression. In humans, ROR1 expression has been reported in several normal adult tissues, including parathyroid glands, pancreatic islets, and regions of the esophagus, stomach, and duodenum [4]. Analysis of thermal stability showed that the melting temperature of DOTA-conjugated ZROR1:A10 was 62 °C, and thus well above body temperature. Importantly, the analysis also demonstrated full refolding after thermal denaturation, which supports the use of macrocyclic chelators for radiolabeling. Such chelators are attractive due to their high thermodynamic stability and kinetic inertness of the radiolabel, but require elevated labeling temperatures [40]. In this study, radiolabeling at 50 °C provided stable labeling (Table 1). *In vitro* specificity tests showed that binding of [^111^In]In-ZROR1:A10 to ROR1-expressing cells was saturable and significantly higher than binding of the non-binding control [^111^In]In-Zcov19s (Fig. 2C, D), demonstrating preserved binding specificity after labeling. Furthermore, affinity determination based on cell-binding data (Fig. 2E) yielded equilibrium dissociation constant values consistent with those determined for binding to recombinant ROR1 using SPR.

*In vivo*, uptake of [^111^In]In-ZROR1:A10 in ROR1-positive MDA-MB-468 xenografts was saturable (Fig. 3A), and markedly higher than the uptake of non-binding control [^111^In]In-Zcov19s (Fig. 4B). Tumor uptake also significantly exceeded that observed in ROR1-negative Ramos xenografts (Fig. 4A). Taken together, these results demonstrate that tumor accumulation of [^111^In]In-ZROR1:A10 is highly ROR1-specific.

In addition to the higher tumor uptake, [^111^In]In-ZROR1:A10 showed somewhat higher uptake than [^111^In]In-Zcov19s in several normal tissues. This difference is likely explained by the low physiological expression of ROR1 in selected murine tissues, resulting in target-mediated uptake of [^111^In]In-ZROR1:A10, whereas Zcov19s recognizes the SARS-CoV-2 Spike protein, which is absent in mice and is not expected to exhibit target-mediated tissue accumulation. Furthermore, differences in amino acid sequence between the two affibody molecules may also contribute to differences in their pharmacokinetic properties and nonspecific tissue distribution.

Results from the dose escalation experiment (Fig. 3A) showed dose-dependent reduction of [^111^In]In-ZROR1:A10 uptake in the tumor and several normal tissues, including the lungs, pancreas, stomach, and segments of the gastrointestinal tract. The decrease in uptake observed with increasing injected protein mass is consistent with a self-blocking effect, likely caused by competition between labeled and unlabeled ZROR1:A10 for accessible ROR1 binding sites. This finding is in agreement with ROR1 expression in pancreatic islet cells, stomach, and duodenum [4]. Importantly, tumor uptake consistently exceeded uptake in these normal tissues, indicating that endogenous ROR1 expression does not prevent specific visualization of ROR1-positive malignancies. This conclusion was further supported by microSPECT/CT imaging (Fig. 6), which clearly visualized ROR1-positive MDA-MB-468 xenografts but not ROR1-negative Ramos xenografts. Importantly, the dose escalation experiment suggested that increasing the injected protein mass beyond 1 µg per mouse (approximately 3 mg/kg of body weight) leads to a significant reduction in tumor-to-organ ratios, and may negatively affect imaging contrast. These findings are relevant for the optimization of protein dose in future translational and clinical studies.

Although ROR1 has historically been described as an oncofetal protein with limited expression in adult tissues, more recent studies have demonstrated low-level cell-surface ROR1 expression in selected normal human tissues [4]. Low levels of soluble ROR1 have also been detected in the serum of healthy individuals (< 200 ng/mL in < 25% of donors) [41]; however, the contribution of circulating ROR1 to tracer distribution remains unclear. These findings highlight the importance of considering physiological ROR1 expression when evaluating ROR1-targeted imaging agents and the potential for on-target, off-tumor effects of ROR1-directed therapies.

Importantly, despite the relatively low B_max_ value determined for MDA-MB-468 cells (approximately 93,000 ZROR1:A10-accessible binding sites per cell), [^111^In]In-ZROR1:A10 showed good retention in tumors over time (Fig. 5), resulting in an increase in tumor-to-blood ratio from 12 ± 2 at 2 h after injection to 17 ± 2 at 4 h after injection.

This contrasts with previously reported studies on short peptide-based ROR1 imaging probes, such as [^68^ Ga]Ga-DOTA-PEG4-PR7 and [^68^ Ga]Ga-DOTA-PEG4-PR3 [22] or [^68^ Ga]Ga-DP1 [23], which showed rapid tumor washout and relatively low tumor-to-blood ratios. Huang et al. reported tumor-to-blood ratios below 1 for [^68^ Ga]Ga-DOTA-PEG4-PR7 and [^68^ Ga]Ga-DOTA-PEG4-PR3 at all evaluated time points up to 60 min post-injection [22]. Similarly, Yang et al. reported tumor-to-blood ratios below 1 and below 2 for [^68^ Ga]Ga-DP1 and [^18^F]AlF-NP1, respectively, at 60 min post-injection [23]. In contrast, [^111^In]In-ZROR1:A10 demonstrated sustained tumor retention and high tumor-to-blood ratios (12 ± 2 at 2 h after injection), supporting the potential of affibody-based targeting for achieving rapid and high-contrast imaging of ROR1-expressing tumors.

At present, no full peer‑reviewed manuscripts have been published describing antibody-based ROR1 imaging probes in sufficient detail. Preliminary data reported in a conference abstract by Artschwager et al. [21] showed that ^89^Zr-labeled anti-ROR1 antibody zilovertamab could specifically visualize MDA-MB-468 xenografts in mice. However, the tumor-to-blood ratio of [^89^Zr]Zr-DFO-zilovertamab was below 4 even at 96 h after injection, indicating substantially lower imaging contrast compared with [^111^In]In-ZROR1:A10 in the same xenograft model.

An important future aspect will be to determine whether ZROR1:A10 recognizes an epitope distinct from those targeted by clinically developed ROR1-directed therapeutics. Although AlphaFold3 modelling predicts binding to the membrane-distal Ig-like domain of ROR1, experimental structural validation will be required to confirm the binding mode. Such information will likely be valuable for the potential use of ZROR1:A10 as a companion imaging agent in patients receiving ROR1-targeted therapy.

## Conclusion

Affibody-based radionuclide imaging of ROR1 expression in malignant tumors is feasible. Further optimization of molecular design and labeling chemistry may enable the development of an imaging probe suitable for non-invasive selection of patients and identification of individuals most likely to benefit from ROR1-targeting therapies.

## Supplementary Information

Below is the link to the electronic supplementary material.

Supplementary file1 (DOCX 1973 KB)

## Data Availability

The datasets generated during and/or analyzed during the current study are available from the corresponding authors on reasonable request.
