# Supplementary material for: Imaging of ROR1 expression in tumors using radiolabeled affibody molecules
Source: Eur J Nucl Med Mol Imaging. 2026 Aug 1;53(13):8104–15. doi: 10.1007/s00259-026-08106-8 (PMC13727809; doi:10.1007/s00259-026-08106-8)
Supplement: Supplementary file 1 [file 259_2026_8106_MOESM1_ESM.docx]

**Supplementary Information**

**Imaging of ROR1 expression in tumors using radiolabeled affibody molecules**

***Affibody production and chelator conjugation***

The DNA sequences of the affibody candidate A10 (hereon referred to as ZROR1:A10) and negative control Zcov19S were subcloned into an *Escherichia coli* expression vector harboring an N-terminal (HE)_3_ tag and a C-terminal cysteine. The subcloning was performed using In-Fusion Snap Assembly (Takara Bio) and the product was transformed to BL21(DE3) *E. coli* cells for expression. Sequences were verified by Sanger sequencing at Eurofins Genomics (Germany). Sequence-verified clones were expressed in *E. coli*, and recombinant proteins were purified from cell lysates through immobilized metal affinity chromatography (IMAC) using HisPur™ Ni-NTA Superflow Agarose (ThermoFisher). Proteins were eluted with 20 mM sodium phosphate, 300 mM sodium chloride, 300 mM imidazole (pH 7.4), followed by buffer exchange to phosphate-buffered saline (PBS, pH 7.4) using PD-10 columns (Cytiva). Purified (HE)_3_-ZROR1:A10-Cys and control were incubated with TCEP in equimolar amounts for 30 min at 37 °C. Maleimide derivative of the DOTA chelator (MedChemExpress) was subsequently added in 10x molar excess and incubated for 2 h at 37 °C. The conjugated proteins were purified using reverse-phase high performance liquid chromatography (RP-HPLC) on a 1200 series HPLC system Zorbax C18 SB column (Agilent Technologies, USA) using Buffer A (0.1 % trifluoroacetic acid) and Buffer B (0.1 % trifluoroacetic acid in acetonitrile). The molecular weights were confirmed by matrix-assisted laser desorption ionization-time of flight (MALDI-TOF) (4800 MALDI-TOF/TOF, Applied Biosystems, ThermoFisher Scientific).

***In silico protein structure predictions***

Protein structure predictions were generated using AlphaFold3 [1] for ZROR1:A10 in complex with the extracellular domain or individual domains of human ROR1 (UniProt: Q01973). Structural models were visualized and analyzed using PyMOL (Schrödinger, LLC).

***Analysis of secondary structure content, thermal stability and refolding***

Lyophilized DOTA-conjugated ZROR1:A10 was resuspended in PBS to a concentration of 0.2 mg/mL and analyzed using a Chirascan Circular Dichroism Spectrometer (Applied Biophysics Ltd, Leatherhead, UK) with a 1 mm path length cuvette. Secondary structure content was assessed by recording ellipticity spectra from 195 to 260 nm and averaging over five scans. Thermal denaturation was subsequently performed by increasing the temperature from 20 °C to 95 °C at a rate of 1 °C/min and monitoring the ellipticity at 221 nm. The thermal melting point (T_m_) was calculated as the inflection point of a four-parameter sigmoidal curve fitted to the temperature-dependent data using GraphPad Prism 10 (Dotmatics, Boston, MA, USA). After cooling the samples to 25 °C, scans were repeated to evaluate refolding.

***Analysis of binding kinetics***

The kinetic evaluation of binding to recombinant ROR1 was performed by surface plasmon resonance (SPR) using a Biacore 8K system (Cytiva, Sweden). A Series S sensor chip CM5 (cat.no. 29149603, Cytiva) was immobilized with 450 RU of recombinant human ROR1 (cat.no. RO1-H5223, Acro Biosystems). DOTA-conjugated ZROR1:A10 and DOTA-conjugated negative control Zcov19S were serially diluted to 20, 4, 0.8 and 0.16 nM in running buffer (PBS with 0.1 % Tween20, pH 7.4). The samples were injected over the chip surface for 120 s, followed by dissociation for 480 s. The surface was regenerated between cycles using a 30 s pulse of 10 mM HCl. The experiment was performed in triplicate and a 1:1 Langmuir curve fitting model was used to estimate kinetic parameters in the Biacore 8K software.

***Screening affibody candidates for mammalian cell binding***

Soluble fusion proteins comprising the affibody candidates ZROR1:A10 or ZROR1:A50 fused to an albumin-binding domain (ABD) [2] were evaluated for binding to ROR1-expressing mammalian cells. MDA-MB-468, HT29, and SKOV3 cells were incubated with 1 μM affibody-ABD fusion protein, and cell-associated binding was analyzed by flow cytometry as previously described [3]. A PE-conjugated anti-human ROR1 monoclonal antibody (clone 2A2, BioLegend, cat. no. 357803) was used as a positive control. At least 20,000 events were acquired for each sample using a Gallios™ flow cytometer (Beckman Coulter, Brea, CA, USA). The laser protocol used for the detection was FL6-660/20 nm for detection of Alexa Fluor 647-HSA, and FL2-575/30 for detection of the PE-labelled antibody controls.

***Radiolabeling and in vitro stability***

For radiolabeling with indium-111, 30-40 μg of DOTA-conjugated ZROR1:A10 or Zcov19s in 0.2 M ammonium acetate (pH 6.3) was incubated with [^111^In]InCl_3_ (6-27 MBq, 10-145 μL 0.05 M HCl) at 50 °C for 60 min. To scavenge unbound indium-111, a 500-fold molar excess of ethylenediaminetetraacetic acid (EDTA) was added, followed by incubation at 50 °C for 5 min. The labeled affibody molecules were purified using NAP-5 size-exclusion columns equilibrated with PBS. Radiochemical yield and purity were determined as previously described [3]. Radiochemical purity was additionally confirmed by radio-HPLC equipped with a radiodetector and a Phenomenex Luna® C18 column (100 Å, 150 × 4.6 mm, 5 μm) with gradient elution of 0.1% TFA in water and acetonitrile at a flow rate of 1 mL/min.

*In vitro* stability was assessed by incubation of the purified radioconjugates with a 1000‑fold molar excess of EDTA in PBS at 37 °C for up to 24 h. Control samples were incubated with PBS alone. Radiochemical purity was analyzed by instant thin‑layer chromatography at 1, 4, and 24 h post‑incubation. All experiments were performed in duplicate.

***Cell culture***

Human breast cancer (MDA‑MB‑468), colorectal cancer (HT29), and ovarian cancer (SKOV3) cell lines were obtained from ATCC (LGC Promochem, Sweden). All cell lines are adherent and reported to express ROR1 [4-7]. Cells were cultured in RPMI‑1640 medium supplemented with L‑glutamine, fetal bovine serum, 100 IU/mL penicillin, and 100 μg/mL streptomycin. Ramos cells, which lack ROR1 expression, were used as an ROR1‑negative control for *in vivo* studies [8].

***In vitro binding specificity, affinity, cellular processing and receptor quantification***

Binding specificity of [^111^In]In-DOTA-ZROR1:A10 was evaluated using a receptor‑blocking assay [9]. Cells were seeded in 6‑well plates (10^6^ cells/well) and incubated with or without non-labeled ZROR1:A10 (1000 nM) at 37 °C for 30 min, followed by incubation with [^111^In]In-DOTA-ZROR1:A10 (1 nM) for 60 min. Cell‑associated radioactivity was collected after medium removal, washing, and alkaline cell lysis, and measured using an automated gamma counter (Wizard 2480). Specific binding was calculated as the percentage of total cell‑associated activity.

Binding specificity of [^111^In]In-DOTA-ZROR1:A10 was additionally evaluated using ROR1-negative Ramos cells in suspension. Ramos cells (approximately 10^6^ cells/tube) were incubated with or without unlabeled ZROR1:A10 (1000 nM) for 30 min at 37 °C, followed by incubation with [^111^In]In-DOTA-ZROR1:A10 (1 nM) for 60 min. MDA-MB-468 cells, used as ROR1-expressing positive controls, were detached with trypsin, collected, and subjected to the same suspension-based binding protocol. Cell-associated radioactivity was measured after washing and normalized to the number of cells.

To evaluate whether the negative control affibody molecule Zcov19s could interfere with ZROR1:A10 binding, cells were pre‑incubated with excess non‑labeled Zcov19s (1000 nM) prior to addition of [^111^In]In-DOTA-ZROR1:A10. Binding of [^111^In]In-DOTA-Zcov19s to ROR1-expressing cells was evaluated in the presence or absence of excess non‑labeled Zcov19s using the same protocol.

Cellular processing and internalization of [^111^In]In-DOTA-ZROR1:A10 (1 nM) were evaluated in MDA-MB-468, HT29, and SKOV3 cells using an acid-wash method [9].

The number of ZROR1:A10-accessible binding sites (B_max_) and affinity (K_D_) were determined as previously described [10]. MDA-MB-468, HT-29, and SKOV3 cells were incubated at 4˚C for 4 h with [^177^Lu]Lu-DOTA-ZROR1:A10 in concentrations ranging from 10 to 20000 pM. Nonspecific binding was determined in the presence of excess unlabeled ZROR1:A10 (3 µM). After incubation, cells were washed, detached, counted, and measured for radioactivity. Data were analyzed using GraphPad Prism software (GraphPad Software).

***Animal studies***

Animal studies were performed in BALB/C nu/nu mice. ROR1‑positive xenografts were established by subcutaneous implantation of MDA‑MB‑468 cells (1 × 10^7^ cells in 100 µL medium) in the right hind leg. As a specificity control, ROR1‑negative Ramos cells (5 × 10^6^ cells in 100 µL medium) were implanted in the left hind leg. Experiments were performed three weeks after tumor implantation, with four animals per data point. Mice were injected intravenously with 60 kBq of radiotracer, and the injected protein mass was adjusted by addition of unlabeled DOTA-ZROR1:A10. To evaluate the impact of injected protein mass on biodistribution and tumor targeting, mice bearing MDA-MB-468 xenografts were injected with 0.3, 1, 3, or 10 µg of [^111^In]In-DOTA-ZROR1:A10 and analyzed at 4 h post‑injection. Targeting specificity was assessed by comparing the biodistribution of [^111^In]In-DOTA-ZROR1:A10 (1 µg) in mice bearing ROR1‑positive MDA‑MB‑468 tumors with mice bearing ROR1‑negative Ramos tumors, and by evaluating the biodistribution of the nonspecific control [^111^In]In-DOTA-Zcov19s (1 µg) in MDA‑MB‑468 xenografts. To assess time‑dependent uptake, biodistribution of [^111^In]In‑DOTA‑ZROR1:A10 (1 µg) was additionally measured at 2 h post‑injection.

SPECT/CT imaging was performed to validate *ex vivo* biodistribution data. One mouse bearing an MDA-MB-468 tumor and one mouse bearing a Ramos tumor were injected with 1 µg (2 MBq) of [^111^In]In-DOTA-ZROR1:A10. At 4 h post‑injection, the animals were euthanized and imaged using a nanoSPECT/CT scanner (Mediso Medical Imaging Systems, Hungary). CT and SPECT acquisitions were performed using standard parameters, and image reconstruction and fusion were carried out using Nucline and TeraTomo software. Images are presented as fused SPECT/CT maximum-intensity projections in coronal and sagittal planes.

**References**

1. Abramson J, Adler J, Dunger J, Evans R, Green T, Pritzel A, et al. Accurate structure prediction of biomolecular interactions with AlphaFold 3. Nature. 2024;630:493–500. doi:10.1038/s41586-024-07487-w.
2. Jonsson A, Dogan J, Herne N, Abrahmsén L, Nygren PA. Engineering of a femtomolar affinity binding protein to human serum albumin. Protein Eng Des Sel. 2008 Aug;21(8):515-27. doi: 10.1093/protein/gzn028.
3. Dahlsson Leitao C, Mestre Borras A, Xu T, Oroujeni M, Liu Y, Westerberg C, et al. Conditionally activated affibody-based prodrug targeting EGFR. J Control Release. 2023;357:185–195. doi: 10.1016/j.jconrel.2023.03.046.
4. Hudecek M, Lupo-Stanghellini MT, Kosasih PL, Sommermeyer D, Jensen MC, Rader C, et al. Receptor affinity and extracellular domain modifications affect tumor recognition by ROR1-specific CAR T cells. Clin Cancer Res. 2013;19:3153–3164. doi: 10.1158/1078-0432.CCR-13-0330.
5. Henry C, Llamosas E, Knipprath-Meszaros A, Schoetzau A, Obermann E, Fuenfschilling M, et al. Targeting the ROR1 and ROR2 receptors in epithelial ovarian cancer inhibits cell migration and invasion. Oncotarget. 2015;6:40310–40326. doi: 10.18632/oncotarget.5643.
6. Tan H, He Q, Gong G, Wang Y, Li J, Wang J, et al. miR-382 inhibits migration and invasion by targeting ROR1 through regulating EMT in ovarian cancer. Int J Oncol. 2016;48:181–190. doi: 10.3892/ijo.2015.3241.
7. Meng S, Li M, Qin L, Lv J, Wu D, Zheng D, et al. The onco-embryonic antigen ROR1 is a target of chimeric antigen T cells for colorectal cancer. Int Immunopharmacol. 2023;121:110402. doi: 10.1016/j.intimp.2023.110402.
8. Baskar S, Wiestner A, Wilson WH, Pastan I, Rader C. Targeting malignant B cells with an immunotoxin against ROR1. MAbs. 2012;4:349–361. doi: 10.4161/mabs.19870. Epub 2012 Apr 26.
9. Yin W, Xu T, Ding H, Zhang J, Bodenko V, Tretyakova MS, et al. Comparison of HER2-targeted affibody conjugates loaded with auristatin- and maytansine-derived drugs. J Control Release. 2023;355:515–527. doi: 10.1016/j.jconrel.2023.02.005.
10. Malmberg J, Tolmachev V, Orlova A. Imaging agents for in vivo molecular profiling of disseminated prostate cancer—targeting EGFR receptors. Int J Oncol. 2011;38:1137–1143. doi: 10.3892/ijo.2011.915.

**Supplementary Table 1. Results from SPR-based screening of affibody molecules binding to human and mouse ROR1.**

Association rate constants (k_a_), dissociation rate constants (k_d_), and equilibrium dissociation constants (K_D_) were estimated for each clone against recombinant human and mouse ROR1.

| **Clone** | **Human ROR1** | | | **Mouse ROR1** | | |
| --- | --- | --- | --- | --- | --- | --- |
|  | *k_a_ (1/Ms)* | *k_d_ (1/s)* | *K_D_ (M)* | *k_a_ (1/Ms)* | *k_d_ (1/s)* | *K_D_ (M)* |
| *A3* | 2.8E+5 | 0.006 | 2.1E-8 | 2.3E+5 | 0.005 | 2.4E-8 |
| *A10* | 4.8E+5 | 0.002 | 4.0E-9 | 9.7E+5 | 0.002 | 2.2E-9 |
| *A32* | 1.7E+5 | 0.004 | 2.1E-8 | 7.7E+5 | 0.006 | 8.2E-9 |
| *A41* | 3.0E+5 | 0.007 | 2.2E-8 | 2.3E+5 | 0.002 | 8.9E-9 |
| *A50* | 3.9E+5 | 0.001 | 2.6E-9 | 3.5E+5 | 0.001 | 2.9E-9 |
| *C4* | 9.3E+5 | 0.026 | 2.8E-8 | 1.3E+6 | 0.012 | 9.1E-9 |
| *C22* | 6.6E+5 | 0.005 | 7.8E-9 | 3.2E+5 | 0.004 | 1.3E-8 |
| *C24* | 4.3E+5 | 0.004 | 1.0E-8 | 2.0E+5 | 0.003 | 1.6E-8 |
| *C30* | 2.4E+5 | 0.015 | 6.3E-8 | 6.2E+5 | 0.029 | 4.8E-8 |

**Supplementary Fig. 1. SPR screening of ROR1 affibody clones against human and mouse ROR1.** Representative SPR sensorgrams showing binding of 11 ROR1-specific affibody clones to immobilized ROR1. (**A**) Human ROR1 and (**B**) mouse ROR1 were immobilized on separate sensor chip surfaces. Each affibody clone was injected at a single concentration of 100 nM. Z_wt_ (non-related IgG-binding affibody) was included as a negative control.

**Supplementary Fig. 2.** **Flow cytometric screening of ROR1-specific affibody binding to human cancer cell lines.** Binding of ZROR1:A10 and ZROR1:A50 to the ROR1-expressing cell lines HT29, SKOV-3, and MDA-MB-468 was analyzed by flow cytometry. Z_wt_ (non-related IgG-binding affibody) was included as a negative control. Cells were incubated with the indicated affibody molecules followed by detection with streptavidin–Alexa Fluor 647 (left and middle panels). An anti-ROR1 antibody (clone 2A2, BioLegend, cat. no. 357803) was included as a positive control together with the corresponding secondary antibody control (right panels). Non-labelled cells and PE-labelled Mouse IgG1, kappa isotype control antibody (clone MOPC-21, BioLegend, cat. no. 400113) were included to assess background fluorescence.

**Supplementary Fig. 3.** Amino acid sequence of ZROR1:A10, including the N-terminal (HE)_3_-tag, the 58 amino acid affibody, and the C-terminal cysteine used for site-specific conjugation of DOTA chelator for subsequent radiolabeling.

**Supplementary Fig. 4.** **Structural models of ZROR1:A10 in complex with ROR1.** (**A**) Predicted complex between ZROR1:A10 and the full extracellular domain (ECD) of ROR1. (**B**) Predicted complex between ZROR1:A10 and the isolated Ig-like domain (IGLD) of ROR1. The IGLD construct corresponds to residues P42–F147 of the UniProt sequence. Structures are colored according to predicted local distance difference test (pLDDT) confidence scores, which reflect per-residue confidence in the predicted structure: dark blue (pLDDT > 90), light blue (90–70), yellow (70–50), and orange (< 50). The predicted interface TM-score (ipTM) and predicted TM-score (pTM) are indicated for each model, where ipTM reflects the confidence in the predicted interface between the binding partners and pTM reflects the confidence in the overall fold of the model.

**Supplementary Table 2. AlphaFold3 modelling of affibody ZROR1:A10 binding to ROR domains**

| **Modelled construct** | **Residues** | **ipTM score** | **Interpretation** |
| --- | --- | --- | --- |
| ROR1 Ig-like domain | 42–147 | 0.87 | High-confidence interaction predicted |
| ROR1 Frizzled CRD | 165–299 | 0.30 | No plausible interaction predicted |
| ROR1 Kringle domain | 312–391 | 0.16 | No plausible interaction predicted |
| ROR2 ECD | 30–400 | 0.19 | No plausible interaction predicted |

**Supplementary Fig. 5.** SDS-PAGE with bands corresponding to purified and DOTA-conjugated candidate ZROR1:A10 and negative control Zcov19S.

**Supplementary Fig. 6.** Mass spectra from MALDI-TOF analysis of candidate ZROR1:A10 (**A**) and negative control Zcov19S (**B**) before and after conjugation to DOTA.

**Supplementary Fig. 7.** SPR sensorgrams showing signals for injection of DOTA-conjugated negative control affibody Zcov19-DOTA at different four concentrations over ECD-ROR1 immobilised on the chip surface.
